# *In vivo* effects of allogeneic mesenchymal stem cells in a rat model of acute ischemic kidney injury

**DOI:** 10.22038/IJBMS.2018.26829.6566

**Published:** 2018-08

**Authors:** Shahrzad Havakhah, Mojtaba Sankian, Gholam Hosein Kazemzadeh, Keyvan Sadri, Hamid Reza Bidkhori, Hojjat Naderi-Meshkin, Alireza Ebrahimzadeh Bideskan, Saeed Niazmand, Ahmad Reza Bahrami, Abolfazl Khajavi Rad

**Affiliations:** 1 Department of Physiology, School of Medicine, Mashhad University of Medical Sciences, Mashhad, Iran; 2 Immunology Research Center, School of Medicine, Mashhad University of Medical Sciences, Mashhad, Iran; 3 Department of Vascular Surgery, Vascular and Endovascular Surgery Research Center, Imam Reza Hospital, Mashhad University of Medical Sciences, Mashhad, Iran; 4 Nuclear Medicine Research Center, Mashhad University of Medical Sciences, Mashhad, Iran; 5 Stem Cell and Regenerative Medicine Research Department, Iranian Academic Center for Education, Culture and Research (ACECR), Mashhad Branch, Mashhad, Iran; 6 Microanatomy Research Center, Mashhad University of Medical Sciences, Mashhad, Iran; 7 Neurogenic Inflammation Research Center, School of Medicine, Mashhad University of Medical Sciences, Mashhad, Iran; 8 Department of Biology, Faculty of Science, Ferdowsi University of Mashhad, Mashhad, Iran

**Keywords:** Acute kidney injury, Acute renal failure Bone marrow-derived mesenchymal stem cell, Cell transplantation Ischemic kidney injury, Rat

## Abstract

**Objective(s):**

Renal ischemia-reperfusion injury (IRI) as a severe condition of acute kidney injury (AKI) is the most common clinical problem with high mortality rates of 35-60% deaths in hospital. Mesenchymal stem cells (MSC) due to unique regenerative characteristics are ideal candidates for the treatment of the ischemic injuries. This work is focused on the administration of MSC to IRI-induced AKI Wistar rats and evaluating their significance in AKI treatment.

**Material and Methods:**

Animals underwent surgical procedure and AKI was induced by 40 min bilateral renal pedicle clamping. Immediately after reperfusion, 2×106 rat bone marrow derived MSCs were injected via intra-parenchymal or intra-aortic route.

**Results:**

Animals subjected to AKI after days 1 and 3 showed significant increase in the serum creatinine and blood urea nitrogen (BUN) concentration along with a declined glomerular filtration rate (GFR) when compared with non-ischemic animals. On the other hand, treated animals showed a significant enhanced regeneration as compared to ischemic animals in both administration route groups.

**Conclusion:**

According to the results concluded from the renoprotective effects of MSC in IRI/AKI, MSCs could be considered as promising therapeutic approach for AKI in clinical applications.

## Introduction

Acute kidney injury (AKI), a heterogeneous clinical syndrome, refers to the progressively kidney failure and declined glomerular filtration rate (GFR) resulting in the accumulation of urea and creatinine in the body. This accumulation of nitrogen wastes results in the impairment of body fluid, electrolytes, and acid-base homeostasis, which is accompanied by significant morbidity and mortality i.e. 23.9% among adults and 13.8% among children. The incidence of AKI among individuals, who have been hospitalized, is significantly greater than the general population (1, 2) and ischemia-reperfusion injury (IRI) has been known as the most common cause of AKI (3). Early diagnosis of AKI is difficult causing irreversible kidney damages in patients, which is being treated with current applied approaches such as dialysis or transplantation (4, 5). Several cell culturing and pre-clinical approaches in animal models are being used to understand the pathophysiology of AKI and finding novel and effective therapeutic strategies (2). Renal IRI is a common experimental model to induce AKI in rat and mice for experimental purposes (2). As per literature, following the induction of renal IRI, the inflammation process is activated and numerous inflammatory mediators such as cytokines, chemokines, and reactive oxygen species (ROS) are secreted and activated, causing recruitment of inflammatory cells for enhanced inflammation leading towards apoptosis and necrosis (4, 6). 

For these reasons, fundamental strategies and cures are needed for the management and treatment of AKI.

Stem cells (SCs) especially mesenchymal stem cells (MSCs) are undifferentiated cells having self-renewal and multi-lineage differentiation capabilities (5-8). These cells have been obtained mainly from bone marrow whereas other sources such as umbilical cord, adipose tissue and other less or non-invasive sources and have potential to treat several diseases, including acute and chronic graft versus host disease, Crohn disease, Sjgren’s syndrome (SS), ulcerative colitis, multiple sclerosis, transplant rejection, systemic sclerosis and systemic lupus erythematous (7). These cells have also been applied in clinics to treat myocardial infarction (10-12), cardiomyopathy, heart failure (13), diabetes (14), osteoarthritis (15), osteogenesis imperfecta, foot and ankle fusion, liver cirrhosis, burn injuries, neurologic disease (16, 17), encephalomyelitis (18), urologic disorders (19), and experimental AKI, as well as, tumor therapy (9). 

In pre-clinical and clinical practices, potential of MSCs to ameliorate renal IRI (20), glycerol model of AKI, cisplatinum model of AKI, glomerulonephritis model (17), Alport’s syndrome, diabetic nephropathy (21), and rhabdomyolysis-associated acute kidney injury (4, 9, 22–25) has been studied comprehensively and it has been concluded that MSCs after their injection, move into ischemic injured kidney tissues and influence parenchymal reconstruction and tissue regeneration, repair injured tubules and improve renal function (4). 

The significant damage of ischemic injury is evidenced in the corticomedullary zone, in particular, S3 segment of the proximal tubule, located in the deep inner cortex and the outer stripe of the outer medulla (2). In spite of the paracrine effects of MSCs, their efficiency to establish a natural cell niche and to regenerate the injury is directly related to the closeness of injury site and administration of the cells. The aim of the present study was to investigate the regenerative potential of MSCs administered immediately after reflow and to compare systemic and locally injection route, in a rat model of IRI/AKI.

## Materials and Methods

***Animals, induction of IRI/AKI, and cell infusions***

All procedures involving animals were approved by the respective Institutional Animal Care of the Mashhad University of Medical Sciences (Mashhad, Iran) and were performed in accordance with the National Institutes of Health Guidelines for the Care and Use of Laboratory Animals. Animals were housed at a constant temperature and humidity, with a 12:12-hr light-dark cycle, and had unrestricted access to a standard diet and tap water. Adult male Wistar rats weighing 250–300 g were used for this study (MUMS, Iran). 

**Figure 1:**
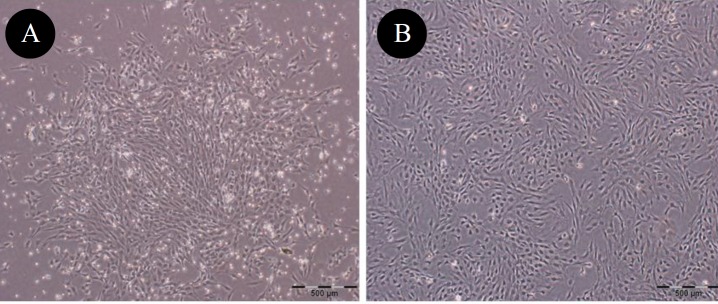
Phase contrast photomicrographs showing morphological characteristics of rat bone marrow-mesenchymal stem cells (BM-MSCs). (A) passage 1, and (B) passage 3. Cells showed spindle -like fibroblastic morphology (×40)

**Figure 2:**
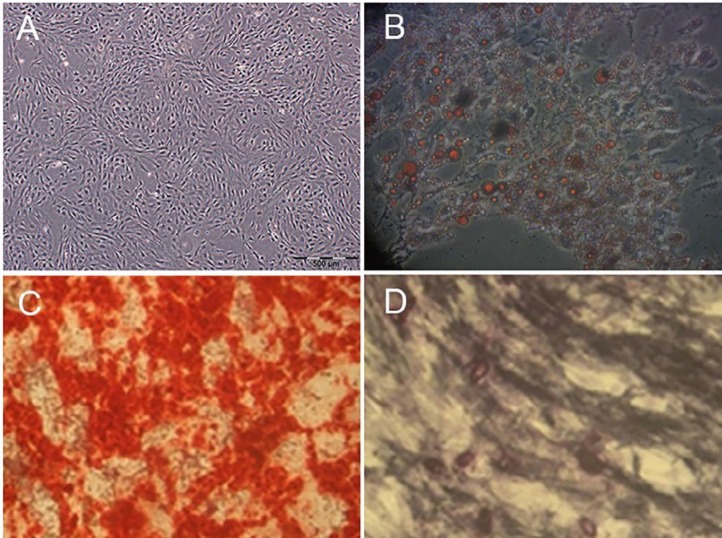
Differentiation characteristics of rat bone marrow-mesenchymal stem cells (BM-MSCs). A: The control sample was cultured in routine medium (×40). B: Adipogenic differentiation was visualized by Oil Red O staining of the lipid vesicles (×200), and C and D: Osteogenic differentiation was detected by Alizarin red staining, and Alkaline phosphatase, respectively (×100)

**Figure 3:**
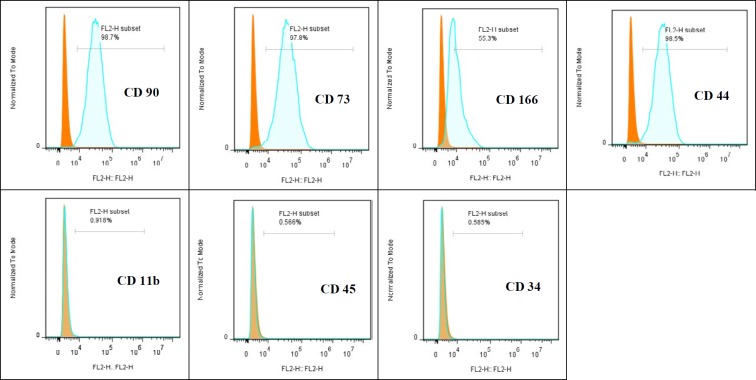
Flowcytometry analysis of rat bone marrow-mesenchymal stem cells (BM-MSCs). Rat BM-MSCs were positive for CD90, CD73, CD166, and CD44, but negative for CD11b, CD45, and CD34 surface markers

**Figure 4:**
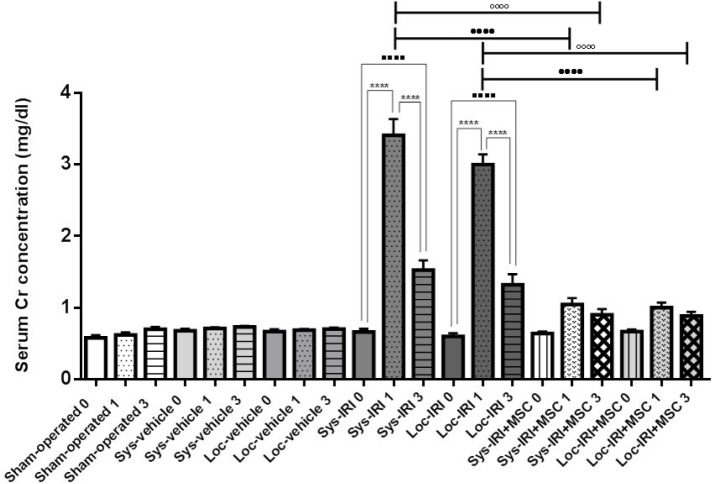
Effect of rat bone marrow-mesenchymal stem cells (BM-MSCs) on serum creatinine following renal ischemia-reperfusion injury (IRI). Serum creatinine was measured at pre-injury (day 0) and 1 and 3 days after surgery. Rats treated with MSCs immediately after reperfusion. **** *P*<0.0001 compared to day 1, ^....^*P*<0.0001 compared to day 0, ••••*P*<0.0001 compared to day 1 in cell treated group (same route injection), and °°°°*P*<0.0001 compared to day 3 in cell treated group (same route injection), (n=5 per group), Cr: creatinine

**Figure 5:**
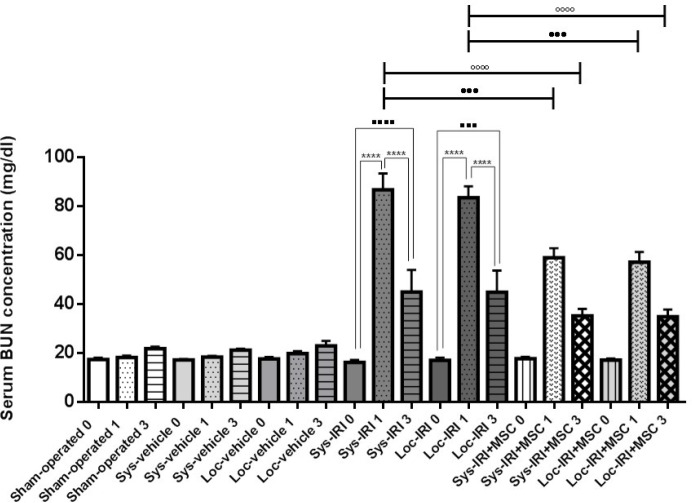
Effect of rat bone marrow-mesenchymal stem cells (BM-MSCs) on serum blood urea nitrogen (BUN) following renal ischemia-reperfusion injury (IRI). Serum BUN was measured at pre-injury (day 0) and 1 and 3 days after surgery. Rats treated with MSCs immediately after reperfusion. **** *P*<0.0001 compared to day 1, ^....^ *P*<0.0001 compared to day 0, ••••*P*<0.0001 compared to day 1 in cell treated group (same route injection), and °°°°*P*<0.0001 compared to day 3 in cell treated group (same route injection), (n=5 per group)

**Figure 6:**
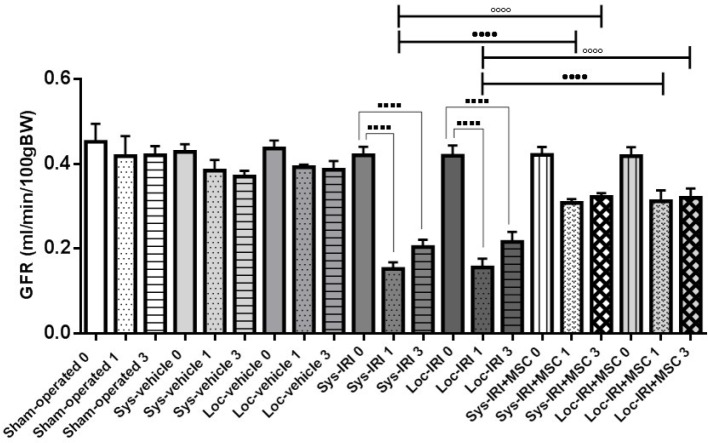
Effect of rat bone marrow-mesenchymal stem cells (BM-MSCs) on glomerular filtration rate (GFR) following renal ischemia-reperfusion injury (IRI). GFR was measured at pre-injury (day 0) and 1 and 3 days after surgery. Rats treated with MSCs immediately after reperfusion. ****P<0.0001 compared to day 0, ••••*P*<0.0001 compared to day 1 in cell treated group (same route injection), and °°°°*P*<0.0001 compared to day 3 in cell treated group (same route injection), (n=5 per group)

**Figure 7:**
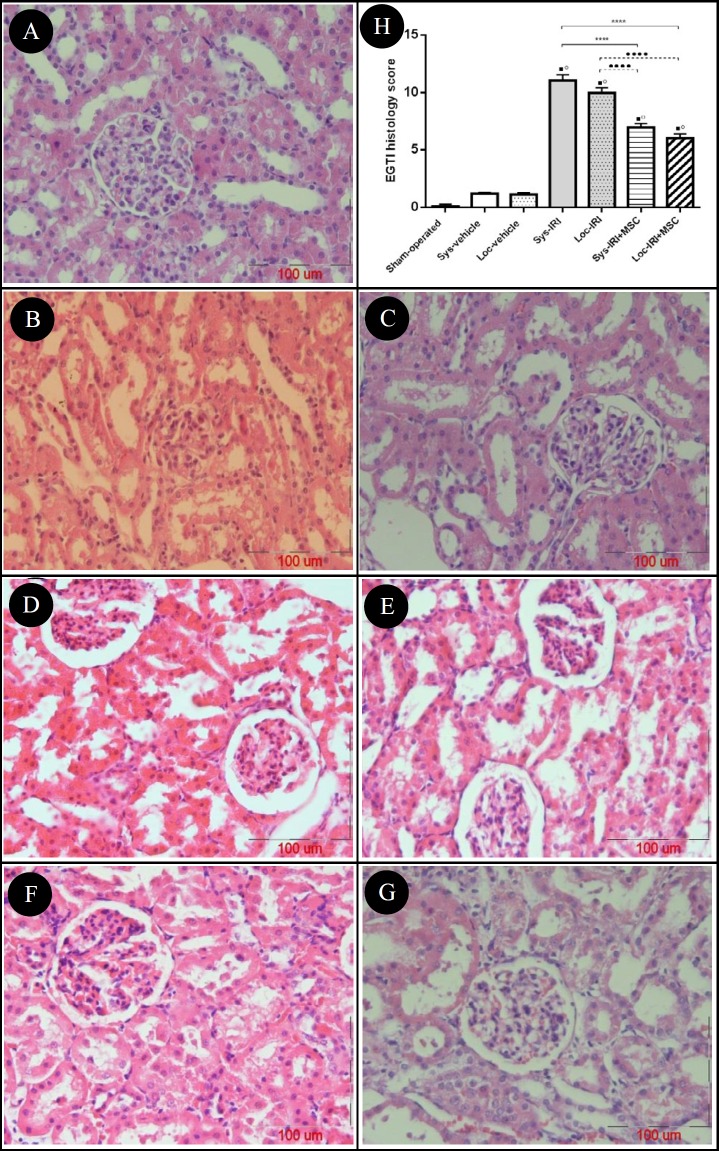
Photomicrograph of pathological changes induced by renal ischemia-reperfusion injury (IRI). The kidney sections were stained by hematoxylin and eosin (H&E) and examined using a light microscope (x400) and scored using the EGTI histology scoring system to assess Endothelial, Tubular, Glomerular and Interstitial cell damage (A-G). Score 0 indicates normal histology, while score 14 displays maximal injury. A: Control (sham operated) group with normal renal morphology, B & C: Sys-vehicle and Loc-vehicle groups with non-significant changes compared with control group. D & E: Sys-IRI and Loc-IRI groups with the distinctive pattern IRI (dilation of renal tubules, cast formation, endothelial loss, as well as tubular epithelial cells necrosis). F & G: Sys-IRI+MSC and Loc-IRI+MSC groups with relatively well-preserved architecture and focal tubular necrosis with lower degree of vacuolization and cast formation. H: Effect of rat bone marrow-mesenchymal stem cells (BM-MSCs) on renal histology following renal IRI. ^.^*P*<0.0001 compared to sham-operated group, °*P*< 0.0001 compared to same route vehicle injection, ****P<0.0001 compared to Sys-IRI group and ••••*P*<0.0001 compared to Loc-IRI group

**Table 1:** Effect of renal ischemia and mesenchymal stem cells (MSC) administration on serum creatinine level

***Groups***	***Day 0***	***Day 1***	***Day 3***
Sham-operated	0.58±0.04	0.62±0.04	0.7±0.03
Systemic vehicle	0.68±0.03	0.71±0.02	0.73±0.01
Local vehicle	0.67±0.03	0.69±0.01	0.7±0.02
Systemic IRI	0.66±0.04	3.41±0.23 ≠ •	1.53±0.14 ≠ •
Local IRI	0.6±0.04	3±0.14 ≠ •	1.32±0.15 … §
Systemic IRI+MSC	0.64±0.02	1.05±0.09 *	0.9±0.08 *
Local IRI+MSC	0.67±0.03	1±0.07 #	0.88±0.06 #

Serum creatinine values (mg/dl) from cell-treated and non-treated groups. There is no significant difference between systemic and local MSC treated animals. Values are mean±SEM.

° *P*<0.0001 compared to day 0 in same group,

* *P*<0.0001 compared to Sys-IRI group, day 1 and,

# *P*<0.0001 compared to Loc-IRI group, day 1,

≠ *P*<0.0001, and

… ‌* P*=0.0002 compared to Sham-operated group in same day, and

• *P*<0.0001, and

§ *P*=0.0003 compared to vehicle-treated group in same route injection and same day

**Table 2:** Effect of renal ischemia and mesenchymal stem cells (MSC) administration on serum blood urea nitrogen (BUN) level

***Groups***	***Day 0***	***Day 1***	***Day 3***
Sham-operated	17.4±1.67	18.2±1.64	21.8±1.79
Systemic vehicle	17.2±0.84	18.34±1.22	21.24±1.26
Local vehicle	17.6±1.82	19.82±2.16	22.94±4.52
Systemic IRI	16.2±2.28	86.8±14.89 ≠ •	44.98±20.02 °… §
Local IRI	17±2.55	83.54±10.49 ≠ •	44.88±19.72 °°— “
Systemic IRI+MSC	17.74±1.67	58.92±8.86 * ≠ •	35.2±6.44 **
Local IRI+MSC	17.1±1.6	57.16±9.3 # ≠ •	34.9±6.47 ##

Serum BUN values (mg/dl) from cell-treated and non-treated groups. Values are mean±SEM.

° *P*<0.0001, and

°° *P*=0.0002 compared to day 0 in same group,

* *P*=0.002, and

** *P*<0.0001 compared to Sys-IRI group, day 1 and,

# *P*=0.0005, and

## *P*<0.0001 compared to Loc-IRI group, day 1,

≠ *P*<0.0001,

… *P*=0.0056, and

— *P*=0.006 compared to Sham-operated group in same day, and

• *P*<0.0001,

§ *P*=0.0037 and,

“ *P*=0.0135 compared to vehicle-treated group in same route injection and same day

**Table 3:** Effect of renal ischemia and mesenchymal stem cells (MSC) administration on glomerular filtration rate (GFR) level

***Groups***	***Day 0***	***Day 1***	***Day 3***
Sham-operated	0.45±0.09	0.42±0.11	0.42±0.05
Systemic vehicle	0.43±0.04	0.38±0.06	0.37±0.03
Local vehicle	0.44±0.04	0.39±0.01	0.39±0.05
Systemic IRI	0.42±0.04	0.15±0.04 ≠ •	0.20±0.04 ≠ §
Local IRI	0.42±0.05	0.16±0.05 ≠ •	0.22±0.05 ≠ “
Systemic IRI+MSC	0.42±0.04	0.31±0.02 *	0.32±0.02 **
Local IRI+MSC	0.42±0.05	0.31±0.06 #	0.32±0.05 ##

GFR values (ml/min/100 gBW) from cell-treated and non-treated groups. There is no significant difference between systemic and local MSC treated animals. Values are mean±SEM.

° *P*<0.0001 compared to day 0 in same group,

* *P*=0.0012, and

** *P*=0.0002 compared to Sys-IRI group, day 1 and,

# *P*=0.0012, and

## *P*=0.0004 compared to Loc-IRI group, day 1,

≠ *P*<0.0001 compared to Sham-operated group in same day, and

• *P*<0.0001,

§ *P*=0.0003, and

“ *P*=0.0002 compared to vehicle-treated group in same route injection and same day

The corticomedullary zone is the best site for locally intraparenchymal injection, so in the absence of the ultrasonic guide, the injection at the proper location would be blind. We studied right and left kidneys of the 40 healthy (control) adult male Wistar rats that were killed in our labs for other experiments. Kidneys were removed and fixed in 10% formalin and dehydrated with different degrees of alcohol and paraffin-embedded blocks were prepared. Two sections (5-micrometer) were prepared from each block and were stained with hematoxylin and eosin (H&E) and the slides were evaluated by morphometric method. To confirm the location of injection site, methylene blue solution was injected in the corticomedullary zone, and then injection site was examined. Thirty gauge needles with length of 1 cm were used, while a micropipette tip of 7 mm was cut from the top and the needle was entered to it, so that 3 mm from the tip of the needle was available for injection.

The rats were randomly assigned to the following groups, each containing of five animals: sham-operated group, systemic/local vehicle treated groups (Sys/Loc-vehicle), IRI with systemic/local vehicle treated groups (Sys/Loc-IRI), and IRI with systemic/local cell treated groups (Sys/Loc-IRI+MSC).

IRI/AKI was induced in pentobarbital-anesthetized animals, and rectal temperature was maintained at 37^°^C, as described elsewhere (23). After a midabdominal laparotomy, kidneys were exposed and renal pedicles were clamped with atraumatic vascular clamps for 40 min. While clamps were applied, in systemic cell/vehicle treated groups, the abdominal aorta was cannulated with IV cannula (gauge 24, O.D. 0.7 mm) for intra-aortic injection. In locally cell/vehicle-treated groups, intraparenchymal injection was performed, as described above. The vehicle in control rats with IRI was infused by the same route. The sham-operated animals underwent similar operative procedures but without any other procedure.

Administration of cells or vehicle was performed immediately after reflow. After visual confirmation of reflow, ~2×10^6^ MSC/animal were given via the supra-renal aorta or intra-parenchymal of kidney (~1×10^6^ MSC/kidney). Control animals for IRI were infused with medium. Aorta puncture site was sutured with 10-0 nylon, covered by fibrin glue and abdominal incision was closed with 4-0 silk, and animals were allowed to recover.

***Cells and culture***

MSCs used for this experiment were generated by standard procedures. Briefly, under sterile conditions 6-weeks old male Wistar rats were killed by chloroform. Then, the femur and tibia were excised and all connective tissues attached to bones were removed immediately (24). Bone marrow plugs were extracted by flushing the bone marrow cavity in sterile PBS, and then the suspension was filtered through 100-µm mesh. The cells were centrifuged, resuspended in complete culture medium containing low-glucose Dulbecco’s modified Eagle’s medium (LG-DMEM; Gipco, USA) supplemented with 10% fetal bovine serum (FBS; Gipco, USA), plated in 75-cm^2^ primary culture flasks (Spl, Life science; Korea), and incubated at 37°C in humidified atmosphere with 5% CO_2_. After 3 days, the medium was changed and nonadherent cells were removed. The MSCs were isolated on the basis of its ability to adhere to the culture flask. At 90% confluence, the cells were trypsinized (0.25% trypsin-EDTA; Gipco, USA) and passaged at 1:3 ratios. The culture medium was changed every 2–3 days. Cells had a typical spindle-shaped appearance, and the MSCs phenotype was confirmed by differentiation into osteocytes and adipocytes with specific differentiation media (25). In addition, superficial markers were negative for CD45, CD11b, CD34 and positive for CD44, CD166, CD73 and CD90 expression, determined by Fluorescence-activated cell sorting (FACS) analysis (28). Passage 3-4 was used in all experiments. The viability of MSC by using trypan blue staining was verified to be >85% before use.

***Biochemical analysis***

Blood samples were collected at baseline and days 1 and 3 post-IRI. Urine samples were obtained from metabolic cage for 24 hr at baseline and days 1 and 3 post-IRI. Creatinine and blood urea nitrogen (BUN) concentration were determined by autoanalyzer (BT3000 Plus, Italy).

***Histology and injury scores***

Kidney tissues were embedded in paraffin and 5 µm tissue sections obtained for H&E staining. Histological damage was scored by the EGTI scoring system. The scoring system consists of histological damage in 4 individual components: Endothelial, Glomerular, Tubular, and Interstitial (score: 0-14). Ten non overlapping fields were randomly chosen from the cortical and corticomedullary junction areas of each section (400 ×magnification) (29). 

***Statistical analysis***

Data are expressed as means±SEM. Primary data collection and statistical analyses were carried out using Prism (GraphPad, San Diego, CA). ANOVA and Bonferoni’s *post hoc* tests were used to assess differences between data. A *P*-value of <0.05 was considered significant.

## Results

Animals were used in this study had a normal blood and urine tests at pre-surgery stage. All animals in control, sham-operated groups and pre-injury day in the treated groups showed a normal kidney function evaluated by serum creatinine and BUN and GFR measurement as well as normal renal histology. 

***Bone marrow -MSCs isolation, culture and characterization***

MSCs, used in the current study, were generated by standard procedures and grown for at least three passages in culture and then morphologically defined by a fibroblast-like appearance (Figure 1). Before *in vivo* usage of MSCs, they were characterized by confirming their ability to undergo osteogenic and adipogenic differentiation (Figure 2) as well as flowcytometry analysis. The cells were negative for CD45, CD11b, and CD34 but positive for the CD44, CD166, CD73 and CD90 superficial markers (Figure 3).

***Intraparenchymal injection***

Histology study of 40 healthy animals revealed that average diameter of kidney cortex in adult male Wistar rat was 2.8 mm (Mean±SEM (2.81±0.7)) as the intraparenchymal injection was performed in 3 mm deep. Due to the limited access to ultrasound guide, injection at the proper location would be blind and we tried our best for a convenient alternative way. While studying the right and left kidneys of 40 healthy (control groups) adult male Wistar rats, we observed 

***Biochemical analysis and functional study***

There were no significant differences between sham-operated group, systemic and local vehicle treated groups in serum creatinine, BUN, and GFR values at any time (Table 1-3). 

Ischemia for 40 min in animals led to severe renal insufficiency, as evidenced by a rise in creatinine, BUN, and decline in GFR values when compared with non-ischemic animals at days 1 and 3.

As shown in Figures 4-6, animals subjected to AKI at 24 hr post-ischemia had significant increase in the levels of serum creatinine to 3.41±0.23 mg/dl (Sys-IRI), 3±0.14 mg/dl (Loc-IRI), and serum BUN to 86.8±6.7 mg/dl (Sys-IRI) and 83.5±4.7 mg/dl (Loc-IRI) when compared with non-ischemic animals (*P*<0.05). The results showed that there are no significant differences between systemic and local ischemic groups at any time (Table 1-3). 

Animals infused with MSC had significantly lower serum creatinine, 1.05±0.09 mg/dl (Sys-IRI+MSC), 1±0.07 mg/dl (Loc-IRI+MSC) (*P*<0.0001, Figure 4), BUN levels 58.92±3.96 mg/dl (*P*=0.0002, Sys-IRI+MSC) and 57.16±4.16 mg/dl (*P*=0.0005, Loc-IRI+MSC) at 24 hr after cell injection compared with same route vehicle-treated IRI animals (Figure 5).

Figure 6 represented the GFR from different treatment groups. A significant increase in the GFR was observed in MSC-treated groups, as compared to those of vehicle-treated IRI animals, Sys- IRI+MSC (0.31±0.01 ml/min/100 gBW) vs. Sys-IRI (0.15±0.02 ml/min/100 gBW) and Loc-IRI+MSC (0.31±0.03 ml/min/100 gBW) vs. Loc-IRI (0.16±0.02 ml/min/100 gBW), (*P*=0.0012, Figure 6) at 24 hr post-ischemia.

***Histological analysis***

We examined the histological characteristics of kidney tissues from each group. Compared with the sham-operated and systemic/local vehicle-treated groups injected with media, there was notable damage in IRI kidneys (Figure 7). When compared with IRI rats, 11±0.5 (Sys-IRI) and 10±0.5 (Loc-IRI), kidneys from MSC-treated rats showed significantly lower degree of EGTI score, 6.98±0.31 and 6.04±0.35 in systemic and local IRI+MSC groups, respectively (Figure 7). 

## Discussion

The present study provides evidence to support that the MSC therapy contributes significant renoprotection in rats with IRI-AKI. Animals infused with MSC immediately after reperfusion had significantly better renal function and lower renal injury scores than untreated animals. This was demonstrated in two different injection route. 

The pathophysiological process after ischemic injury causes several functional and structural changes in renal tissue involving cellular metabolism, cytoskeleton, extra cellular matrix generation and activation of inflammatory factors such as cytokines, chemokines, and ROS molecules (30). 

Despite these changes, surviving renal tubule cells have a remarkable ability to regenerate and proliferate after ischemic AKI. As our results showed, untreated animals had a relative recovery in renal function 72 hr after ischemia.

Cell-based regenerative therapeutic applications are being considered as the most promising approaches to treat several diseases including IRI/AKI (11, 31–34). Cellular mechanisms such as transdifferentiation, de-differentiation or homing of SC, paracrine or endocrine effects of administered SC and expansion/proliferation of resident stem/progenitor cell play critical role in the regenerative capabilities of injured tissues (30). The protective effects via differentiation-independent mechanisms, is due to cytokines secretion and increased expression of growth factors such as HGF, VEGF, and IGF-I, or their anti-apoptotic and mitogenic properties (3, 4, 22, 35–38). Significance of these paracrine effects of MSCs in regenerative medicine has gained much attention of scientists for IV injection of cells. In spite of such paracrine effects, the best administration route for the most effectiveness of stem cells is to be close to the site of injury and natural cell niche. It has been shown that the effect of cell injection at the site of injury in some tissues is much better than systemic administration. Several studies have focused on the local injection of the cells close to the injury site in ischemic heart and brain i.e. in many myocardial infarction models, direct injection of stem cells at the center of infarct and border zone areas of the myocardium is more effective than systemic injection (11, 31–34). 

It is well known that renal ischemia usually damages the corticomedullary zone. The S3 segment represents the remainder of the proximal tubule, located in the deep inner cortex and the outer stripe of the outer medulla (2). Because of that, the corticomedullary zone is the best site for locally intraparenchymal injection. In one study, cells were injected by hydrogel to increase homing in the tissue (39). In other two studies, the cells were injected into the corticomedullary zone under ultrasound guide (40, 41). 

As mentioned, the corticomedullary zone is the best site for intraparenchymal injection, so in the absence of the ultrasonic guide, the injection at the proper location was blind. We studied the 80 healthy kidneys and achieved cortical diameter.

The integration percentage of administrated stem cells is usually below 1% in any given organ. On the other hand, MSCs are known to have immunomodulatory properties and secrete various growth factors and cytokines, which may explain further possible mechanisms including inhibition of fibrosis and apoptosis, improvement of angiogenesis, stimulation of mitosis, proliferation and differentiation of intrinsic stem cells (42-45). The kidney can recover after damage following repolarization, cell de-differentiation, migration, and proliferation and the release of growth factors, chemokines and cytokines can facilitate repair process (30). 

The administration of MSC, hematopoietic stem cell (HSC), or a bone marrow transplant, following glycerol-, cis-platinum, or IRI/AKI, protected kidney tissue and improved renal function in rodents (3, 44). As with other studies (3, 44), animals infused with MSC, either systemic or local injection, compared with untreated MSC animals, had significantly better renal function as early as 24 hr following reflow (3, 4), which was determined by decreasing the Cr level approximately 33%.

In accordance with comparative analysis of literature, it was observed that administration of MSC, independently to the administration route, significantly improved renal function at days 1 and 3 after induction of IRI/AKI among animals treated by MSCs. 

The mechanism behind the regeneration could be transdifferentiation of MSCs and differentiation-independent protective effects such as cytokines secretion and increased expression of growth factors such as hepatocyte growth factor (HGF), vascular endothelial growth factor (VEGF), and insulin-like growth factor 1 (IGF-I), or their anti-apoptotic and mitogenic properties. Pathological data was enough to justify the paracrine effects in MSC-based kidney regeneration (3, 4, 22, 35–38). As discussed by several other studies, the infusion of MSC into the suprarenal aorta in IRI/AKI model has no adverse effects like respiratory and renal problems or death (4, 44). Moreover, the advantage of local injection is directly trapping into the injury site. Results of the current work demonstrate that local injection has slightly better effect than intra-aortic injection but these differences are not statistically significant. 

## Conclusion

Our data showed the promising kidney-protective effect of MSC in the rat IRI/AKI experimental model employing both intra-aorta and intra-parenchymal injection routes. Administration of MSCs significantly improved renal function and histopathologic indices at days 1 and 3 after induction of IRI/AKI. These effects at least in part, mediated by complex paracrine actions that are able to significantly protect and regenerate the damaged kidneys. More investigations are required to determine the mechanism responsible for this recovery. 
